# The Impact of Microwave Drying on the Structure of Exemplary Soils—Insights Using X-ray Microtomography

**DOI:** 10.3390/ma15175891

**Published:** 2022-08-26

**Authors:** Łukasz Kaczmarek, Małgorzata Jastrzębska, Tomasz Wejrzanowski

**Affiliations:** 1 Faculty of Building Services, Hydro and Environmental Engineering, Warsaw University of Technology, Nowowiejska 20, 00-653 Warsaw, Poland; 2 Faculty of Civil Engineering, Silesian University of Technology, Akademicka 5, 44-100 Gliwice, Poland; 3 Faculty of Materials Science and Engineering, Warsaw University of Technology, Wołoska 141, 02-507 Warsaw, Poland

**Keywords:** drying of soil, microwave heating, soil structure, computed microtomography, water content

## Abstract

In the field of soil drying methods, rapid microwave heating is progressively replacing conventional techniques. Due to the specific heat transport caused by microwaves, the drying process can significantly modify soil structure, which, in turn, can influence mechanical and filtration characteristics. In this study, we compared structural changes of exemplary non-cohesive (medium quartz sand (MSa)) and cohesive soil (silty clay mainly composed of kaolinite (siCl)). The sample materials were subjected to three different drying methods: air-drying, conventional oven (CO) drying, and microwave oven (MO) drying (MO). Soil structure was studied using X-ray microtomography (XµCT) and described in detail by image analysis methods. The study showed that the analyzed types of heating had a negligible effect on the structure of the sands, but a significant impact in the case of silty clay. Such a phenomenon is discussed and explained in this paper. The study advances the testing of soils microwave drying in a geotechnical laboratory.

## 1. Introduction

Different soil drying methods can have various effects on the structure and, thus, on the geomechanical and filtration features (thermally induced thermo-hydro-mechanical behavior of saturated or unsaturated soils [1,2,3]). The rapid change in conditions can affects the soil fabric [4] and therefore, it can weaken the soil grains or solid material, leading to cracking, fracturing, and crushing. Furthermore, with cohesive soils, the repeated drying procedure may cause bond degradation or cementation. Moreover, temperature variations can initiate mineralogical changes. All these phenomena can also occur in situ, although on a different time scale and markedly smaller temperature ranges (generally to approximately 40 °C [4,5]). 

Many experimental studies have explored rapid microwave drying [6,7] and the influence of multiple wet–dry cycles (such as those that occurred during laboratory drying) on the physical and mechanical properties of soil or its mixtures with various additives (e.g., with cement, liquid modifiers, bentonite, etc.), which include thermally modified swelling parameters [8,9], hydraulic conductivity/permeability coefficients [8,10,11], shear strength [12,13], durability, stiffness, and the void ratio [12], Atterberg limits [8], and even intensification of landslides [11,14,15]. Listed soil features which may be (but do not have to be) affected by the impact of thermal changes are strongly related to changes in the macrostructure (crack intensity factor, length, and cracks opening), the microstructure (e.g., porosity and density), and the geochemical composition (mineralogy and chemical composition) of the soils [10]. Nevertheless, reducing the drying time of soils has many implications, which make the procedure of accelerated drying of soils in the laboratory and their potential field applications important research issues.

Therefore, in this pilot study, the focus is on the influence of rapid changes in water content (because of microwave drying) on the structure of selected soil (specifically, medium sand and hydrophobic silty clay). The impact of temperature on soil structure is a complex phenomenon and requires a multifaceted approach, especially due to the variety of existing microwave drying procedures. In order to evaluate the effects of microwave drying, comparative drying in a conventional oven and air drying were performed. The reference test was the air-dry state of a sample that was not exposed to high-temperature drying. Non-invasive and non-destructive X-ray computed microtomography (XµCT) was used to determine structural changes. 

## 2. Materials and Methods

The test procedures involved three types of drying tests using soil samples, after which the samples were exposed to X-ray computed microtomography. Structurally similar sand (3) and clay (3) samples were used in this study. The three types of drying methods used were: air-drying (21 °C, 48 h), drying in a conventional oven (105 °C, 24 h), and drying in a microwave oven (800 W, 7 min, estimated temperature < 200 °C for sand and <400 °C for silty clay). 

The air-drying method was the reference test because of its non-invasive characteristic (drying at room temperature). The second method used heat energy (conventional oven drying), and is a well-standardized method for soil testing. The third method (the microwave radiation drying method) seemed to be a fast and effective alternative as compared with the previous method.

### 2.1. Selected Soils and Their Preparation

This comparative study tested two exemplary soil types (cohesive and non-cohesive): medium sand (MSa) and silty clay (siCl). The exact types were determined based on granulation curves according to the European standard classification [16]. According to the Unified Soil Classification System (USCS), MSa is poorly graded clean sand (SP) and siCl is inorganic clay (CL). Quartz is the dominant component of the sandy material, and silty clay is mainly composed of kaolinite. These two materials were subjected to three drying methods: one sample from each specific soil type per drying method. The range of tests was designed to indicate possible directions of study with regards to changes in structure, for the purpose of future, more focused testing.

**Figure 1:**
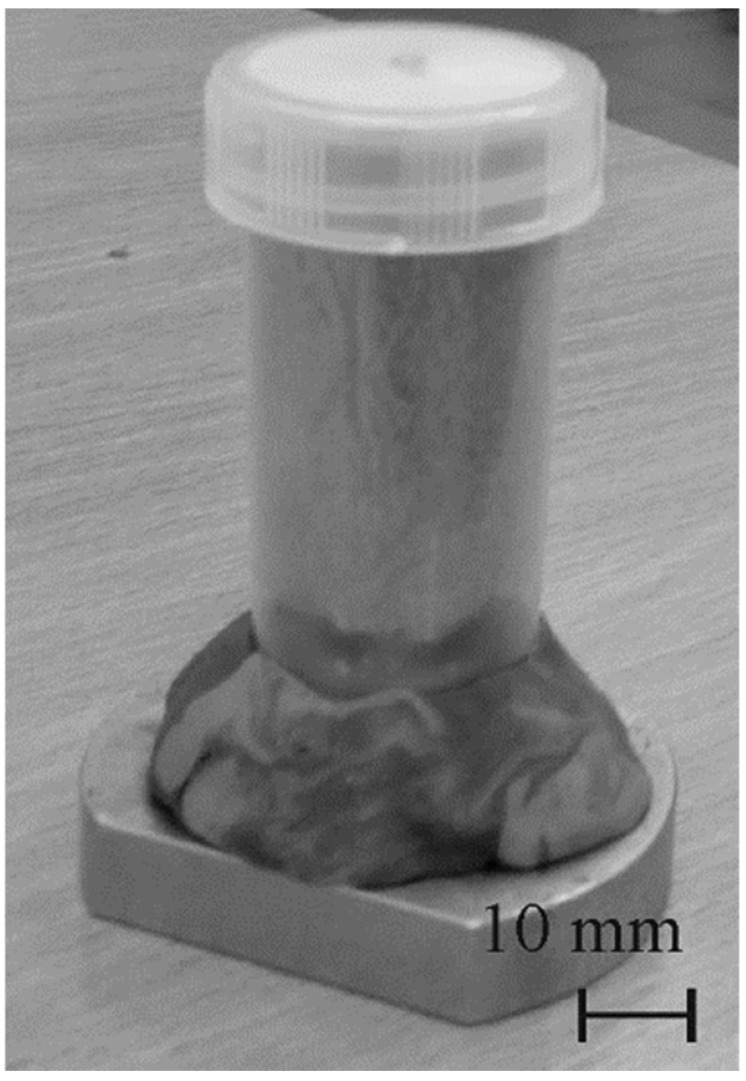
A medium sand sample prepared for testing in microtomography.

The selection of appropriate containers for the samples was very important. Materials must be X-ray transparent and resistant to heat, as the temperature increase of some cohesive soils exposed to microwave energy can exceed 200 °C, reaching up to 800 °C [17]. For this purpose, specially designed polypropylene rigid cylindrical transparent containers (Figure 1) were chosen, measuring 25 mm in diameter, 50 mm high, and with a high melting point (>400 °C). These containers are resistant to heat energy and microwave radiation and can be placed directly into the microtomograph after drying. The density of the material lent rigidity and strength to the containers, but the density had to be lower than the soil density to avoid disturbing the microtomographic images. Figure 1 shows a container filled with a sandy sample.

The medium sand originated from excavations at a highway construction site in southern Poland. Its average water content in natural state (in situ state) was just a few percent (w ≈ 3%). The characteristic diameter d_50_ for the prepared soil samples was 0.42–0.47 mm. Its basic material characteristics are given in Table 1. Due to the loose state of the soil in the field, sandy material was carefully piled up to fill the entire container without additional compaction. Sample weights without containers were approximately 48 g.

The silty clay came from the Porcelain Factory in Tułowice, in southern Poland. The experimental material was macroscopically homogeneous. The range of its water content in the natural state (in situ state) was 20% to 30%. Its basic material characteristics are given in Table 2. Due to the stiff state of the soil in the field, the sample could be cut out from a larger silty clay fragment, and then trimmed by the container during careful pushing. Sample weights, without containers, were approximately 55 g. According to a literature study [18], it can be stated that the lack of colloidal activity (low value of Skempton’s coefficient A ~0.56) confirmed significant dominant presence of kaolinite in the mineral composition.

### 2.2. Procedure of Soil Drying by Air, Oven, and Microwave

Generally, different soil drying methods were basically used to calculate the elementary physical soil parameter of the water content. The water (moisture) content in the soil can be expressed as:

(1) $$w=100\cdot \frac{M_{w}}{M_{s}}$$

where *w* is the water content (%); *M_w_* is the mass of water in soil (g), that is, the difference between the mass of the wet sample (initial mass, *M_i_* (g)) and the mass of the sample after drying (final, dry mass, *M_s_* (g)); *M_s_* is the dry mass of soil (g).

Drying of soil samples according to standards such as PKN-CEN ISO/TS 17892: 2009 [20] or ASTM D 2216-19 [21] is typically carried out in a conventional oven for at least 24 h at a temperature of 105–110 °C. The soils can also be dried using microwave radiation. However, in this case, the procedure was not as clear-cut. This type of soil drying has been the subject of research for many years and so far, only in the United States (ASTM D4643-00 [22] and ASTM D4643-08 [23]), Australia (AS 1289.2.1.4-2015 [24] and AS 1289.0:2014 [25]), and France (NF P 94-049-1 [26]), standardized guidelines of different levels of detail have been published regarding the determination of soil moisture in microwave ovens. In addition, many national reports have been created as a result of research, for example, in Canada (ATT 15/96 [27]) or Hong Kong (Chung and Ho’s report from 2008 [28]). However, it is worth noting that the indicated guidelines do not take into account all variants related to soil heterogeneity and research methods. The selection of the specific heating power and the mass of the specimen are still, generally, arbitrary decisions of the researcher. Numerous examples can be found in Jastrzębska’s work [6].

In this study, the soil samples were subjected to air, oven, or microwave drying methods. For all samples, the same type of container was used. After drying, the containers were secured with a screw cap to prevent changes in water content and spillage, and then taken for X-ray testing.

Reference air-drying tests were performed in the laboratory, where an air-conditioning unit ensured a steady room temperature (~21 °C) and air humidity (~50%). The durations of these tests were 48 h. The second group of two samples was dried (according to [29]) in a conventional oven (CO) at 105 ± 5 °C in a traditional laboratory drier with convection drying. The time duration for these two tests was 24 h. 

The third sample group was dried in an 800 W microwave oven (MO), model WD800AP20-6, which corresponded to the most probable temperature increase, up to 200 °C (cohesionless material) and 400 °C (hydrophobic cohesive material), based on estimations from [17,30,31]. Higher temperatures are less possible due to no large quantities of adsorbed water; specific susceptible minerals (allophane or other hydrophilic materials, which are more prone to temperature rise than study hydrophobic kaolinite); or excessive organic content. Therefore, there was no cause for soil specimen dehydration, and the samples could not catch fire, or develop dangerously high temperatures (1000 °C) [17]. Aspects related to the variability of the temperature, soil preparation, and technical solutions complicate the possibility of evaluating the temperature resulting from microwaves. In the research, at this stage, microwave power was used to describe the methodology as a temperature driver. Such an approach is also related to the common use of microwave power for a description of drying procedures. Nonetheless, this is a very important issue, which is worth more detailed discussion.

There is no universal drying procedure with microwave heating. It is used for a wide variety of purposes (e.g., preparation for further specialist research, to reduce swelling [32,33], and to clean polluted soils [34,35]) and for a wide variety of soil types, including high inhomogeneity of microstructure even within the same soil sample, that is, with various mineralogical compositions and structures. Nevertheless, it is believed that microwave drying can be applied for all types of soils, excluding soils with organic matter content exceeding 10% and bentonites with water content higher than 110% [36], as well as soils with a high content of halloysite, mica, montmorillonite, gypsum, and other hydrated materials [28].

The microwave oven used for the soil drying process was mainly adjusted to: soil type, sample mass and number, spatial arrangement, and drying time. The selection of soil mass and drying time strictly correlated with the heating power of the device. Care should be taken to avoid explosion or burning of dried soils (e.g., clay soils and fine sand, gravel particles, and hard brittle rock, especially when they have a high water content [6,17,28,37]). General recommendations include using 700–800 W microwave power and 2–50 minute drying time intervals. Details can be found, among others, in studies by [6,36,38].

For the concerned cases in this study, the following experimental procedures were performed: The first two mass measurements during microwave drying were performed at intervals of 30 s, and then every 1 min until a constant mass was obtained. For both MSa and siCl, the total drying time was 7 min. In this study, the limitation on sample mass (approximately 50 g) was dictated by the size-related requirements affecting the duration and quality of XµCT results.

### 2.3. X-ray Computed Microtomography

X-ray CT is an imaging technique followed to observe the internal structure of objects. By rotating the X-ray source and detector, a series of projections of the sample can be generated. The contrast of the projection image is a function of the absorption coefficient, which is determined by the density of the material. In the case of complex soils, the absorption coefficient is correlated with the sum of all chemical components and moisture of the material [39]. The studied samples were dried, hence, during the XµCT exposure the registered change of absorption coefficient was related mainly to material density. 

Among other methods, microtomography has been successfully used to assess the qualitative and quantitative characteristics of sandy and clay soil structure (e.g., [40,41] studied water-induced structure changes with use of XµCT). As part of this study, XµCT was also used to analyze the soil structure characteristics, but in relation to three drying approaches. Thus, after drying, all 6 dried samples were taken to a desiccator for cooling. Then, the samples were sequentially put on a so-called holder, and finally placed in the tomograph cell. An XµCT analyzer and the Data Viewer software were employed to analyze the outcome images, while the Avizo Fire software was used to develop numerical models of samples. Qualitative descriptions included structural variability and the differentiation of grains. Three-dimensional numerical modeling of the samples was used to produce precise analyses of the span of fractures and the distribution of voids. The results of spatial analyses of the sample models were used for the calculation of the porosity parameter (including fractures and volume of void space), average pore diameter (i.e., the sum of all pores or free spaces diameters divided by their number), centroid path tortuosity (ratio of the real length of the flow path to the straight line between the start and the end of flow), and equivalent diameter; the parameters have been described in [42]. 

Microtomography was provided with an air-cooled Hamamatsu L8121-03 X-ray tube (with tungsten anode) that generated conical X-rays. The tests were carried out with an 8 second exposure time of a single radiograph, at a voltage of 120 kV and power of 10 W. A single spatial image of the sample (consisting of 1601 radiographs) required a test duration of approximately 4 h. At the selected technical exposure settings, the minimum size of the voxel side was 28 microns (i.e., 28 × 28 × 28 microns cube), which was the smallest identifiable element of structure. The raw images originating during the scanning were converted to an 8-bit digital form with a resolution of 1024 × 1024 pixels. Reconstructed tomographic images are presented on a shades of gray scale (higher density zones are lighter).

The study question about the effects generated by various drying methods was, “Are there any effects?”; therefore, as a reference, the tomographic results of air-dried samples were used. There were no tomographic exposures of the samples before drying because the procedure of filling the samples was controlled; no larger voids, loosening, or linear discontinuities were observed. The accuracy of the acquired images was sufficient to identify significant changes. The structural elements below the voxel size remained ”masked”, and, to identify them, the SEM technique is recommended. However, this technique also has its limitations, i.e., small area of recognition and surface recognition, as well as, in the case of soil, quite complex preparation of research material. Therefore, for this study, we focused on the greatest and unambiguous effects of high temperature on soil samples.

## 3. Results and Discussion

The water content test results of the study samples (Table 3) are typical for these soils occurring in the field, at the places of collection. The average water content of the medium sand was ~3.2% with a standard deviation of 0.22%. Due to the large pores and ready evaporation of humidity, such variation in results can be considered to be a natural variation of the research material. The average water content of the silty clay was ~22.5% with a standard deviation of 0.65%. Slightly lower water content was associated with air drying (a difference of up to 1.6%), which could be associated with water present in closed pores. Thus, the use of different methods and different drying times produced similar effects, militating in favor of the shortest method.

The similar water content values confirm the estimated (based on the results of similar tests on similar materials [17,30,31]) temperature range associated with drying in MO. Such test results indicated the absence of dehydration processes (expected to be just above 400 °C in the case of kaolinite, dehydration curve [17]), which would result in higher water content values in microwave tests than in other methods with lower temperatures.

**Figure 2:**
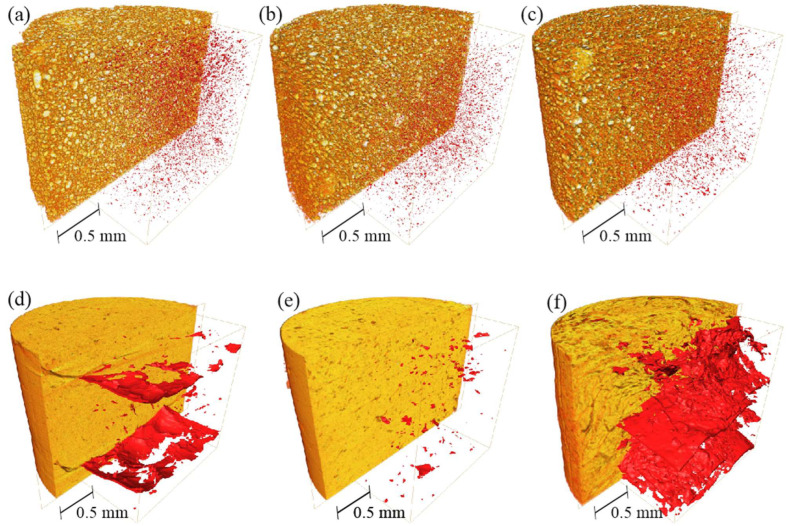
3D images of dried sand and silty clay: in air without heating (**a**,**d**); in a conventional oven (**b**,**e**); in a microwave oven (**c**,**f**). Pores and fractures are shown in red.

Digital overall spatial microstructure reconstructions of sand and silty clay images after drying in air, in a conventional oven, and in a microwave oven are presented in Figure 2.

The structure of the sand is heterogeneous and consists of uniformly dispersed grains and pores (shown in red). In the silty clay, air drying produced few fractures, CO drying produced some voids and small fractures, and MO drying produced major fractures (Figure 2a). 

**Figure 3:**
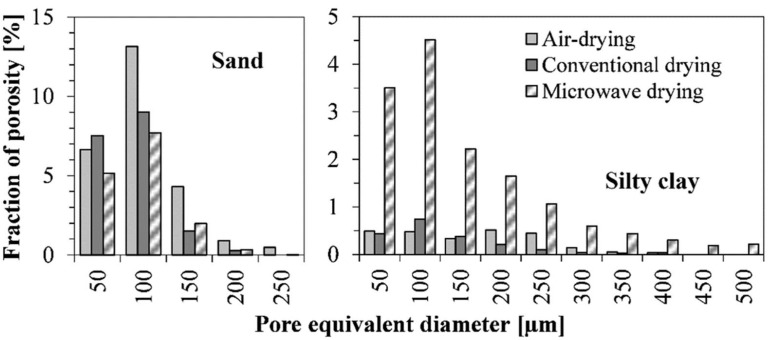
Pore volume fraction by equivalent diameter.

Three-dimensional images of the structures were quantitatively analyzed and the outcomes are summarized in Table 4. Notably, XµCT identified structure components bigger than the voxel size in the reconstructed 3D images. Therefore, the measured porosity may be smaller than the ”true” porosity, as pores that are too small cannot be seen. Moreover, according to Table 2 and Jastrzębska’s tests [43], the silty clay samples have a void ratio of *e* = 0.71 (based on the formula *e* = (*RD* − *DBD*)/*DBD*, where *RD* is relative density and *DBD* is dry-bulk density), which corresponds to *n* = 42% porosity (based on the formula *n* = *e*/(1 + *e*)), whereas the measured (by XµCT) porosity is in the range 2–15% (Table 4). The 27–40% difference means that most voids have a diameter of less than 28 µm. The same applies to other parameters in Table 4 and especially Figure 3 since, once again, they probably differ from the ”true” values. In addition, the largest structural elements have the greatest impact on soil behavior.

The results show that the porosity of the sand structure decreases when the drying process is performed at elevated temperatures. Nevertheless, the fraction of pores is similar for the samples heated conventionally and with microwaves. Other parameters determined for sands, such as average pore size or centroid path tortuosity, do not reveal relevant differences. 

The results of the quantitative analysis of silty clay structure show that microwave drying causes significant soil fracturing. In this case, the pore volume fraction, which is mainly represented by cracks, increased significantly (6–7 times higher) as compared with the same parameter for conventionally dried or air-dried samples. 

In all the samples, the average pore size and pore size distribution (see Figure 3) are similar.

After the general spatial structure analysis presented above, sand grains, in turn, merit closer attention: The fractions and basic parameters are set out below in Table 5.

**Figure 4:**
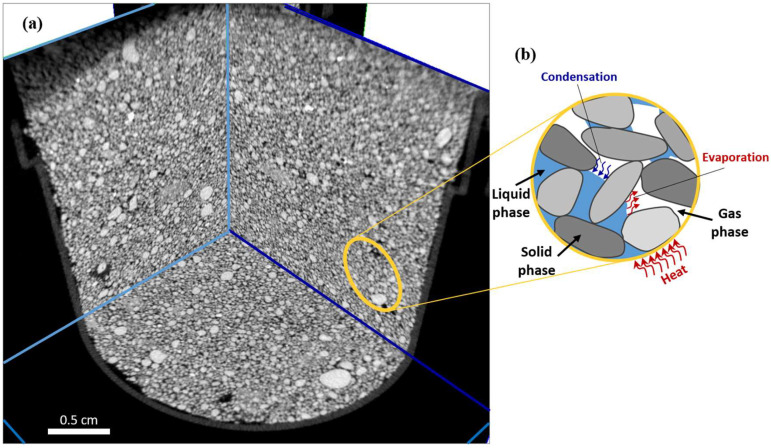
Sand microwave drying: perpendicular microtomographic sections through the medium sand sample after microwave drying (**a**); schematic of unsaturated soil illustrating moisture evaporation and condensation modified from [1] (**b**).

The content of the largest fraction is the highest in the sample not subjected to a rapid temperature rise. This is reflected in the uniformity coefficient. However, the differences are marginal (up to 0.23%). Markedly larger differences could indicate the crushing and cracking of grains of larger fractions under the influence of temperature. However, no cracks were found in the analyzed samples (Figure 4). Grains are enrobed and have a continuous character. Certainly, this is influenced by the dominant share of tough quartz and low humidity, and hence, the limited effect of microwave radiation. For fine quartz sands with high water content (above 23%), the phenomenon of microwave drying has a completely different nature and can even lead to the explosion of samples resulting from too much evaporation (tests conducted on samples of 25 g [6]). For this reason, it is worth expanding the range of samples with higher water content and determining critical saturation in subsequent tests by using XµCT. 

Thus, the weak effect of the drying process on sandy soil can be explained by the fact that any stress that might accumulate during heating (Figure 4b) is released by the motion of sand grains bonded through water by weak mechanical/physical forces. The motion of the sand grains might be responsible for the compacted structure of the sand sample, and therefore, the reduced porosity as compared with the air-dried sand. With respect to this, an important aspect of the grain interaction process that occurs is its contact points, which are involved in load transfer [44].

**Figure 5:**
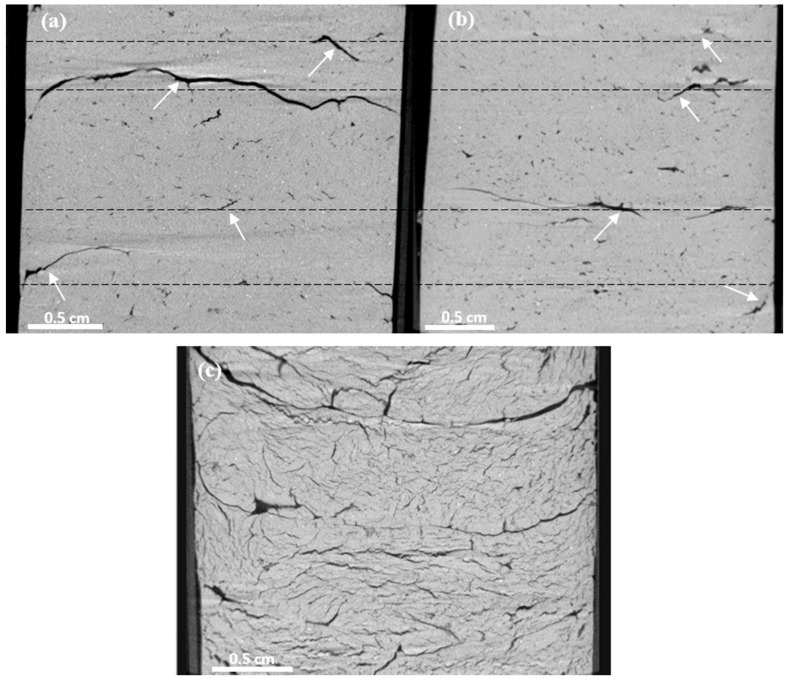
Perpendicular microtomographic sections through silty clay samples after drying by various methods (arrows indicate the similar exemplary fracture locations): air drying (**a**); conventional oven (**b**); microwave (**c**).

In the case of the silty clay tests, in relation to the effects of drying that we discussed earlier, it is worth considering the causes. Hence, convectional drying is an air-to-air method in which hot dry air is the factor that transfers heat to the soil and removes moisture. Air flows around the samples in a natural way similar to air drying where the temperature is much lower but the mechanism in CO drying is the same. Conventional drying by convection means that heat is delivered from outside through the surface of the material, which explains why the surface has the highest temperature, which is the reason why the fracture contours are formed parallel to the surface in cylindrical probes. Warm air has the strongest effect on the upper surface of the samples; therefore, the upper part of the sample dries, and then shrinks, resulting in the final formation of an extensive longitudinal fracture (Figure 5). Apparently, a high and steady temperature in CO drying results in overall homogeneous heating of the sample, and thus, causes less cracking than air drying. However, some zoning could be observed in the tested samples subjected to air drying and CO drying, where the gaps in both cases developed or started to appear in similar places. The structure of the tested air-drying and CO drying samples had the aforementioned numerous smaller voids and gaps (Figure 4 and Figure 5) that may constitute places for the facilitated development of subsequent extensive fractures.

The different mechanisms of water–soil interactions during MO drying in the tested silty clay (which is mainly composed of kaolinite) result in the drying process exerting a marked effect on the structure of the material. In this case, water is chemically bonded to the soil and, when it is removed, the local properties of the material are changed. Furthermore, the pressure accumulated by the evaporating water cannot be released only by cracking, as all soil structure elements are ”cemented”. Thus, during microwave drying, heating is associated with electromagnetic radiation, which induces vibrations of water molecules in the sample, forming an internal source of heat. Hence, microwaves have a greater impact on specimens with higher water content (as well as lower permeability). This observation corresponded to results of tests by [1], who noted the possibility of remarkable sensitivity of the thermo-hydro-mechanical response to a change in temperature in “unsaturated clay, while this observation is absent in soils whose permeability is greater than that of silty sand”. In this way, the microwaves penetrate the interior of the material, heating up the entire volume from the inside. This internal penetration can be observed in Figure 5c as the result of MO heating. There is a system of parallel fractures (parallel to the upper sample surface), with smaller perpendicular secondary fractures growing from the larger fracture and using the smaller fractures and pores to form a background system.

Fracture growth at the lab scale might have a negative effect on the properties identified in static loading (e.g., triaxial shearing [45]). Zones with such fractures in the field scale can be used as privilege slip surfaces [46,47]. Furthermore, a high number of fractures significantly increases the permeability to water and may cause suffusion of fine particles within the soil.

The processes and effects that accompany heating due to CO and MO drying bring about some practical findings. First, MO drying, unlike the time-consuming CO drying, cannot be used ad hoc. The long drying time associated with CO drying allows the soil structure to adapt to the changing conditions of the water content. For this reason, before using the MO drying method, a literature analysis should be conducted regarding the recommended technical parameters of MO drying and its procedure. When there is no recommendation, preliminary research of specific soils should be performed. 

The results of these studies have shown that, despite the various structural degradations caused by CO and MO drying, both drying techniques can be considered to be alternatives to the estimation of soil water content (see Table 2). However, when pre-drying is the early step for further testing, these methods cannot be used interchangeably, especially when drying of cohesive soils is considered (see Figure 5). In remolded cohesive samples dried using MO drying, the presence of rapidly formed discontinuities initially weaken the soil material. When soil is dried too rapidly, it can also cause the occurrence of overheated and stiffened fragments that consequently cause falsification of the resultant parameters describing the soil compressibility or elasticity ( most likely an increase in the compressibility modulus or Young’s modulus values). Furthermore, the characteristics of filtration and consolidation will change: filtration may increase (owing to the better permeability of the fracture system), but consolidation may ”decrease” (a stiffer, dry medium consolidates faster). Therefore, the MO drying method is not recommended for cohesive soils tested, for example, in a direct shear or a triaxial compression apparatus.

In the case of non-cohesive soils, the results indicate that the appropriate MO drying procedure marginalizes the undesirable possible effects of rapid and intense heating. This conclusion has been supported by studies that have been conducted on a larger number of non-cohesive samples [6,7]. Furthermore, the greater the fraction of non-cohesive soil, the faster the drying can be performed, which is important and helpful in preparing samples several times from the same material, for example, direct shear tests. However, the mineral skeleton may weaken due to frequent high-temperature applications. Consequently, premature grain crushing and further reductions in strength parameters (such as the angle of internal friction) may occur, for example, in triaxial tests (especially when applying higher effective stresses) or dynamic triaxial tests. Nevertheless, for example, according to unpublished data, in the case of cohesive soils, a clear increase in the angle of internal friction by approximately 25–40% is observed. Geotechnical design based on such overstated parameters may lead to a construction disaster (failure). In the case of non-cohesive soils, the changes are negligible, in the order of 1%.

## 4. Conclusions

This comparative research investigated the effect of selected drying procedures on the structure of exemplary soils, i.e., medium quartz sand (MSa) and silty clay (siCl) mainly composed of kaolinite; both types of soil were subjected to air drying, drying in a conventional oven (CO), and microwave drying (MO). The structures after drying were qualitatively and quantitatively described by analysis of 3D images obtained by X-ray computed microtomography (XµCT). 

The most breakthrough deliverables of this study are:

- Silty clay, as a cohesive soil, is significantly more sensitive to drying processes than the non-cohesive sand sample. The results obtained in this research showed severe fracturing of silty clay for MO-dried samples. In the case of sandy material, MO drying was successfully applied. The grains of the samples with relatively low water content used in the study did not crack. Thus, the heating type (CO or MO) is not interchangeable in the presented case of the selected cohesive soil. Further studies with different water contents combined with XµCT analyses are recommended.
- Uncritical use of MO drying in laboratories (due to the significant reduction in drying time) may result in incorrectly determining the mechanical and filtration parameters of the soils. The impact of microwaves on the soil during the study of basic physical properties, and then using the same soil for testing shear strength or water permeability, can cause a change in these characteristics. In the case of their reduction, they are underestimated, and in the case of their increase, they are overestimated. As a result, this may cause additional costs associated with the desire to unnecessarily improve their specific characteristics, or there may be a risk of construction failure.
- The drying method had a considerable effect on the porosity of the treated samples. After CO drying, the porosity was 28% lower than after air drying. In MO drying, porosity was even 40% lower than after air drying. This is related to the soil skeleton response to rapidly evaporating water.

## Figures and Tables

**Table 1:** Basic physical properties and classification characteristics of medium sand (MSa—SP) from southern Poland.

Parameter	Symbol	Unit	Value
Specific gravity	ρ_s_	g/cm^3^	2.65
Effective diameter	d_10_	mm	0.20
	d_30_	mm	0.34
	d_50_	mm	0.42
	d_60_	mm	0.48
	d_90_	mm	1.15
Coefficient of uniformity	C_u_	-	2.40
Coefficient of curvature	C_c_	-	1.20
Clay fraction	ClF	%	0
Silt-size fraction	SiF	%	0.38
Sand fraction	SaF	%	99.54
Gravel fraction	GrF	%	0.08

**Table 2:** Basic physical properties and classification characteristics of Tułowice silty clay (siCl—CL, modified from [19]).

Parameter	Symbol	Unit	Value
Specific gravity	ρ_s_	g/cm^3^	2.64
Liquid limit	LL	%	42.2
Plastic limit	LP	%	20.0
Plasticity index	PI	%	22.2
Liquidity index	PL	-	0.60–0.78
Activity	A	-	0.52–0.60
Effective diameter	d_10_	mm	0.0001
	d_30_	mm	0.001
	d_50_	mm	0.0046
	d_60_	mm	0.008
	d_90_	mm	0.05
Coefficient of uniformity	C_u_	-	73
Coefficient of curvature	C_c_	-	1.25
Clay fraction	ClF	%	37
Silt fraction	SiF	%	55

**Table 3:** Results of the water content tests.

Soil Type	Water Content (%) by		
	Air Drying	Drying in a Conventional Oven	Drying in a Microwave
Quartz medium sand	3.1	3.5	3.0
Silty clay (mainly consisting of kaolinite)	21.7	23.3	22.6

**Table 4:** Quantitative characteristics of the microstructure of soils dried by various methods.

Soil	Parameter	Type of Drying		
		Air	Conventional	Microwave
Sand	Porosity (%)	25.5	18.3	15.2
	Average pore diameter (µm)	95.2	98.5	92.3
	Centroid path tortuosity	1.42	1.67	1.85
Silty clay	Porosity (%)	2.50	2.00	14.70
	Average pore diameter (µm)	120.3	111.2	106.3
	Centroid path tortuosity	8.25	8.92	2.70

**Table 5:** Basic properties of medium sand (MSa—SP).

Parameter	Symbol	Unit	Soil Air Dried	Soil Dried in a Conventional Oven (CO)	Soil Dried in a Microwave Oven (MO)
Clay-size fraction	ClF	%	0.04	0.38	0.28
Silt-size fraction	SiF	%	0.00	0.00	0.00
Sand-size fraction	SaF	%	99.65	99.54	99.49
Gravel-size fraction	GrF	%	0.31	0.08	0.23
Coefficient of uniformity	C_u_	-	1.68	2.40	2.29
Coefficient of curvature	C_c_	-	1.04	1.20	1.15

## Data Availability

Not applicable.
